# Disturbance of Fatty Acid Metabolism Promoted Vascular Endothelial Cell Senescence via Acetyl-CoA-Induced Protein Acetylation Modification

**DOI:** 10.1155/2022/1198607

**Published:** 2022-08-10

**Authors:** Tong Lin, Wan-qi Yang, Wen-wei Luo, Li-li Zhang, Yan-qi Mai, Zi-qing Li, Si-tong Liu, Lu-jing Jiang, Pei-qing Liu, Zhuo-ming Li

**Affiliations:** ^1^Laboratory of Pharmacology and Toxicology, School of Pharmaceutical Sciences, National-Local Joint Engineering Laboratory of Druggability and New Drugs Evaluation, Guangdong Province Engineering Laboratory for Druggability and New Drug Evaluation, Guangdong Provincial Key Laboratory of New Drug Design and Evaluation, Sun Yat-sen University, Guangzhou, China; ^2^Department of Pharmacy, Guangdong Provincial People's Hospital, Guangdong Academy of Medical Sciences, Guangzhou 510080, China

## Abstract

Endothelial cell senescence is the main risk factor contributing to vascular dysfunction and the progression of aging-related cardiovascular diseases. However, the relationship between endothelial cell metabolism and endothelial senescence remains unclear. The present study provides novel insight into fatty acid metabolism in the regulation of endothelial senescence. In the replicative senescence model and H_2_O_2_-induced premature senescence model of primary cultured human umbilical vein endothelial cells (HUVECs), fatty acid oxidation (FAO) was suppressed and fatty acid profile was disturbed, accompanied by downregulation of proteins associated with fatty acid uptake and mitochondrial entry, in particular the FAO rate-limiting enzyme carnitine palmitoyl transferase 1A (CPT1A). Impairment of fatty acid metabolism by silencing CPT1A or CPT1A inhibitor etomoxir facilitated the development of endothelial senescence, as implied by the increase of p53, p21, and senescence-associated *β*-galactosidase, as well as the decrease of EdU-positive proliferating cells. In the contrary, rescue of FAO by overexpression of CPT1A or supplement of short chain fatty acids (SCFAs) acetate and propionate ameliorated endothelial senescence. *In vivo*, treatment of acetate for 4 weeks lowered the blood pressure and alleviated the senescence-related phenotypes in aortas of Ang II-infused mice. Mechanistically, fatty acid metabolism regulates endothelial senescence via acetyl-coenzyme A (acetyl-CoA), as implied by the observations that suppression of acetyl-CoA production using the inhibitor of ATP citrate lyase NDI-091143 accelerated senescence of HUVECs and that supplementation of acetyl-CoA prevented H_2_O_2_-induced endothelial senescence. Deficiency of acetyl-CoA resulted in alteration of acetylated protein profiles which are associated with cell metabolism and cell cycle. These findings thus suggest that improvement of fatty acid metabolism might ameliorate endothelial senescence-associated cardiovascular diseases.

## 1. Introduction

Vascular aging accelerates functional and structural deterioration in the vascular system and contributes to the pathogenesis of a majority of age-related diseases including hypertension, atherosclerosis, and vascular cognitive impairment [1–3]. Vascular aging is initiated by endothelial cell senescence [1–3]. Under chronic exposure to cardiovascular risk factors, the endothelial cells undergo a premature senescent phenotype that is characterized by cellular senescence and growth arrest, oxidative stress, excessive release of inflammatory factors, and impairment of endothelium-dependent vasorelaxation [4, 5]. Currently, the pathophysiological mechanisms of endothelial senescence are not fully understood. Exploration of the cellular and molecular mechanisms of endothelial senescence may pave the way for therapeutic intervention for vascular aging-related diseases.

Recently, pathophysiological significance of endothelial cell metabolism has been receiving growing attention. Endothelial cell metabolism, including glycolysis, fatty acid metabolism, and amino acid metabolism, plays a pivotal role in the regulation of endothelial cell proliferation and angiogenesis [6–8], endothelial activation [9], endothelial hyperpermeability [10], redox homeostasis [11], and endothelial-to-mesenchymal transition (EndoMT) [12]. Disturbance of endothelial cell metabolism accelerates the development of various vascular diseases, such as atherosclerosis [13], pulmonary hypertension [14], and cancer [6, 8].

Although endothelial cells primarily rely on glycolysis for energy production [6], fatty acid metabolism gains increasing interest following the discovery that fatty acid oxidation- (FAO-) derived acetyl-coenzyme A (acetyl-CoA) and nicotinamide adenine dinucleotide phosphate (NADPH) are involved in maintaining the homeostasis of endothelial cell. Endothelial cells metabolize fatty acids to acetyl-CoA, which is a critical modulator of EndoMT through restraining TGF-*β*-Smad signaling by acetylating and stabilizing the inhibitory Smad7 [12]. Moreover, endothelial FAO sustains the tricarboxylic acid (TCA) cycle for redox homeostasis through NADPH regeneration, therefore regulating endothelial activation and endothelial hyperpermeability [11]. Furthermore, FAO in endothelial cell regulates biomass synthesis and facilitates deoxynucleotide triphosphate (dNTP) production required for DNA synthesis during endothelial cell proliferation [7].

In view of the importance of fatty acid metabolism in modulating endothelial homeostasis [15–18], the present study was designed to investigate the changes of fatty acid metabolism in senescent endothelial cells and the potential regulatory role of fatty acid metabolism in endothelial senescence, in order to provide therapeutic strategy targeting endothelial cell metabolism for vascular aging and related cardiovascular diseases.

## 2. Results

### 2.1. Endothelial Senescence Was Accompanied by Repression of Fatty Acid Metabolism

**Figure 1:**
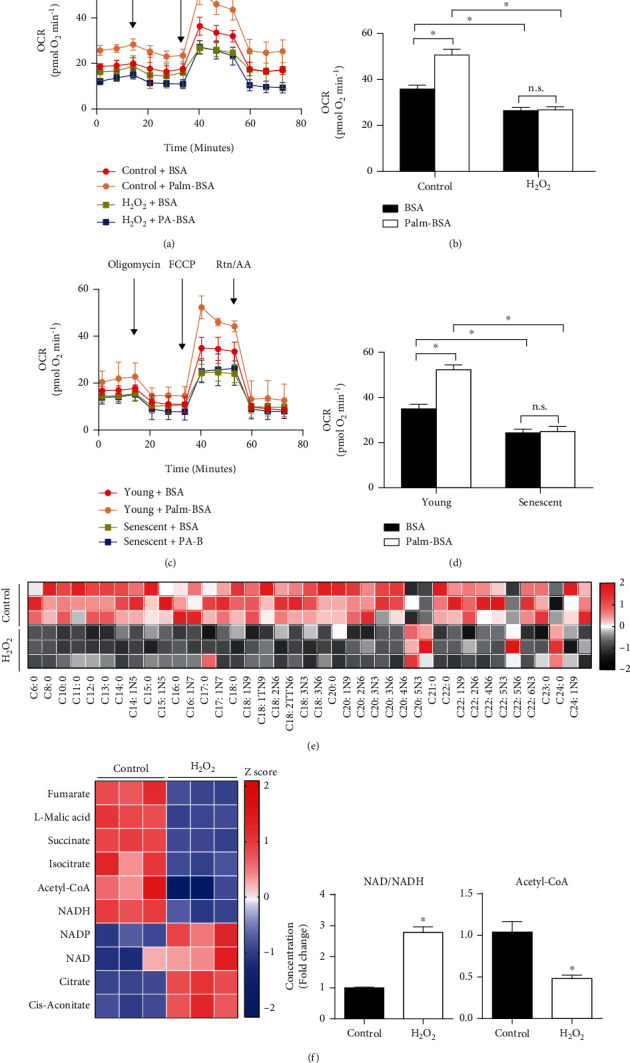
Repression of fatty acid metabolism was observed in senescent endothelial cells. (a) Oxygen-consumption rate (OCR) was measured by XF96 extracellular flux analyzer in HUVECs stimulated with or without H_2_O_2_ and palmitate conjugated bovine serum albumin (Palm-BSA). (b) Bar chart showing the capacity of FAO as determined by analyzing the respiratory capacity of OCR (*n* = 5). (c) OCR and Palm-BSA-induced OCR were investigated by XF96 extracellular flux analyzer in senescent endothelial cells (p13-16). (d) Bar chart showing the capacity of FAO as determined by analyzing the respiratory capacity of OCR in young or senescent HUVECs (*n* = 5). Data were presented as means ± SEM. ^#^*P* < 0.05 vs. control BSA and ^∗^*P* < 0.05 vs. control/young. (e) Fatty acid profile was measured by GC/MS in HUVEC senescence model induced by H_2_O_2_. *n* = 3. (f) TCA cycle metabolites were measured by LC/MS in HUVEC senescence model induced by H_2_O_2_. Ratio of NAD to NADH as well as level of acetyl-CoA was calculated. *n* = 3. Data were presented as means ± SEM. ^∗^*P* < 0.05 vs. control.

In this study, fatty acid metabolism was studied by examining the palmitate-based oxygen-consumption rate (OCR) in both H_2_O_2_-induced endothelial senescence and replicative endothelial senescence induced by population doublings in cell culture. OCR was significantly elevated by stimulation of palmitate-conjugated bovine serum albumin (Palm-BSA) in the control cells or young endothelial cells. In contrast, OCR was suppressed in H_2_O_2_-induced senescent cells and in late-passage cells with or without Palm-BSA treatment (Figures 1(a)–1(d)). These observations reveal a decline in fatty acid metabolism during endothelial senescence. In addition, fatty acid profile was investigated in oxidative stress-induced endothelial senescence. The results demonstrated that levels of most medium and long chain fatty acids (MCFAs and LCFAs) were reduced in senescent endothelial cells; both saturated fatty acids and unsaturated fatty acids including mono-unsaturated fatty acids and poly-unsaturated fatty acids were decreased; methyl linoleate and docosahexaenoic acid (DHA), which are protective to endothelial cell function [19], were declined in senescent endothelial cells as well (Figure 1(e)). Moreover, endothelial senescence was accompanied by a fall in TCA cycle-associated organic acid metabolites, including fumarate, L-malic acid, succinate, and isocitrate, as well as in acetyl-CoA, an important metabolic product of FAO; meanwhile, a rise in intracellular NAD/NADH was observed in endothelial senescence (Figure 1(f)). Taken together, these results suggest that disorder of fatty acid metabolism is involved in endothelial senescence.

### 2.2. Level of Enzymes Associated with Fatty Acid Metabolism Was Altered in Senescent Endothelial Cells

To explore the possible mechanisms underlying the abnormality of fatty acid metabolism, mRNA expressions of proteins associated with fatty acid uptake, transport, and beta-oxidation in senescent endothelial cells were investigated. Among the proteins involved in fatty acid transmembrane transport and activation in endothelial cells [20], including fatty acid transport protein (FATP) 6, the pm, 4 and 5 subtypes of fatty acid-binding protein (FABP), as well as type 3 and 4 acyl-CoA synthetase of LCFAs (ACSL), FABP4 and ACSL3 were remarkably downregulated in both H_2_O_2_-induced and replicative endothelial senescence models (Figure S1A). Additionally, proteins responsible for fatty acid import into mitochondria, including carnitine palmitoyl transferase 1 (CPT1) located in the outer mitochondrial membrane and carnitine palmitoyl transferase 2 (CPT2) located in the inner mitochondrial membrane [21], were significantly decreased (Figure S1B). On the contrary, the mRNA expression of enzymes related to fatty acid oxidation including mitochondrial trifunctional protein subunit ɑ/*β* (HADHA and HADHB), long/medium/short chain 3S-hydroxyacyl-CoA dehydrogenase, enoyl-CoA hydratase, or 3-hydroxyacyl-CoA dehydrogenase (HADH) was slightly altered, although some of these enzymes showed a decreased trend in one of the endothelial senescence models (Figures S1C and S1D). These observations suggest that downregulation of proteins associated with fatty acid uptake and mitochondrial entry might probably contribute to the repression of fatty acid metabolism in endothelial senescence.

**Figure 2:**
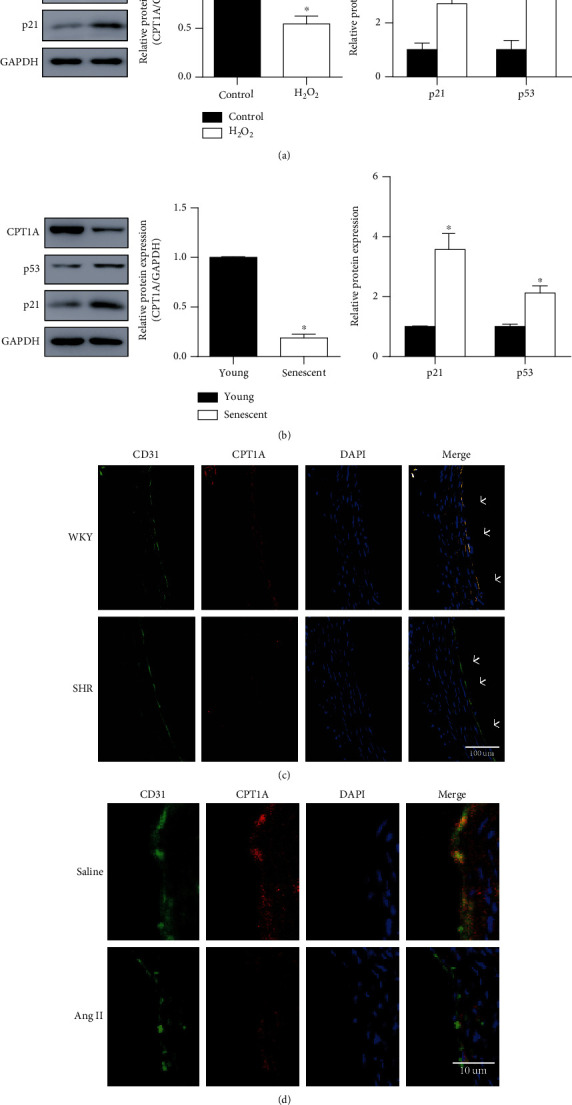
CPT1A expression was downregulated during endothelial senescence *in vitro* and *in vivo*. CPT1A protein expression was measured by Western blot in (a) H_2_O_2_-induced senescent endothelial cells and (b) replicative senescent endothelial cells. *n* = 3. Data were presented as means ± SEM. ^∗^*P* < 0.05 vs. control/young. Immunofluorescent staining of CPT1A was performed in the frozen aortic sections of (c) SHRs and their normotensive control WKY rats and (d) mice infused with or without Ang II for 4 weeks. CD31 represented the endothelial layer. DAPI represents cell nucleus of the vasculature. Merge of CD31 (green) and CPT1A (red) was shown in yellow and indicated the expression of CPT1A in the endothelial layer. *n* = 4.

CPT1, the rate-limiting enzyme of FAO, contains 3 isoforms: CPT1A, CPT1B, and CPT1C [11]. Among these isoforms, CPT1A was the most abundant one expressed in the endothelial cells (Figure S2). The protein expression of CPT1A was studied in the senescent endothelial cell models. The expression of CPT1A was diminished accompanied by the upregulation of senescent markers p53 and p21, confirming that CPT1A was repressed in endothelial senescence (Figures 2(a) and 2(b)). Moreover, the endothelial expression of CPT1A was determined in the aortas of two typical animal models with endothelial senescence, the spontaneously hypertensive rat (SHR) [22] (Figure S3) and Ang II-infused mice [23, 24]. As compared to their normotensive controls, the expression of CPT1A in the endothelial layer of aortas was obviously diminished in SHR and Ang II-infused mice (Figures 2(c) and 2(d)), consistently with the in vitro results.

### 2.3. CPT1A-Dependent FAO Protected against Endothelial Cell Senescence

**Figure 3:**
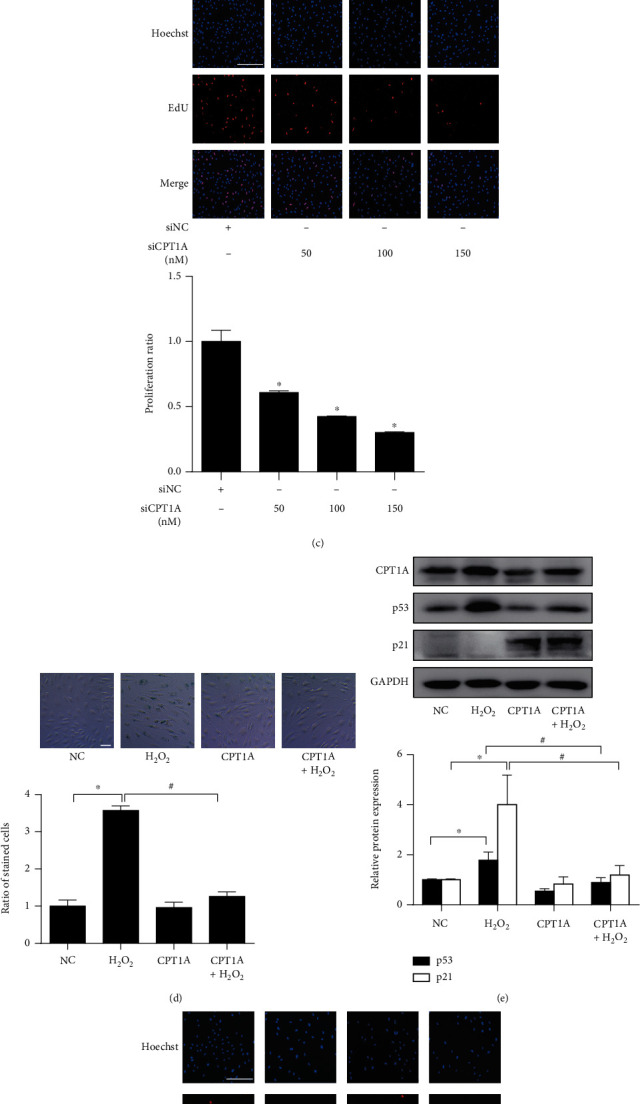
CPT1A-dependent FAO protected against endothelial cell senescence. (a) SA-*β*-gal staining (scale bar: 50 *μ*m), (b) Western blot showing the protein expression of p53 and p21, and (c) EdU staining showing the ratio of proliferating endothelial cells (scale bar: 100 *μ*m), were performed in HUVECs treated with different concentrations of CPT1A siRNA or nontargeted siRNA control (siNC). *n* = 3. Data were presented as means ± SEM. ^∗^*P* < 0.05 vs. siNC. (d) SA-*β*-gal staining, (e) the protein expression of p53 and p21, and (f) EdU staining were investigated in HUVECs transfected with or without CPT1A plasmid, in the presence or absence of H_2_O_2_ stimulation. *n* = 4 ~ 5. Data were presented as means ± SEM. ^∗^*P* < 0.05 vs. control; and ^#^*P* < 0.05 vs. H_2_O_2_.

Since CPT1 catalyzes the rate-limiting step of converting acyl-coenzyme A into acyl-carnitines, which can be transported across mitochondria membranes, and since CPT1A is the most abundant isoform, CPT1A is regarded as the rate-limiting enzyme of LCFA oxidation in endothelial cells [11]. The fall of CPT1A in senescent endothelial cells thus prompted the hypothesis that suppression of CPT1A-dependent FAO might lead to the development of endothelial senescence. To test this hypothesis, the effect of CPT1A knockdown by siRNA or inhibition by etomoxir (ETO) on endothelial cell senescence was investigated. Among the four tested CPT1A siRNAs, siRNA-1 demonstrated the best efficiency and was thus selected for the following studies (Figure S4). CPT1A siRNA-1 augmented the portion of senescence-associated-*β*-galactosidase- (SA-*β*-gal-) positive senescent cells (Figure 3(a)), increased the expressions of cell cycle repressors p53 and p21 in endothelial cells (Figure 3(b)), and attenuated the ratio of EdU-positive proliferating cells in a concentration-dependent manner (Figure 3(c)). Similarly, treatment of CPT1 pharmacological inhibitor ETO [25] facilitated endothelial senescence dose and time dependently (Figure S5).

Moreover, the effect of CPT1A overexpression on H_2_O_2_-induced endothelial senescence was examined by transfecting a Flag-labeled CPT1A plasmid, which remarkably upregulated CPT1A in HUVECs (Figure S6). Overexpression of CPT1A repressed the increase of SA-*β*-gal-positive cells (Figure 3(d)), increased expression of p53 and p21 caused by H_2_O_2_ (Figure 3(e)), and reversed H_2_O_2_-induced arrest of cell proliferation (Figure 3(f)), thus suggesting that CPT1A ameliorates oxidative stress-induced endothelial senescence.

Taken in conjunction, these observations indicate that CPT1A-dependent LCFA metabolism could protect against endothelial senescence. Suppression of CPT1A might probably contribute to the development of endothelial senescence.

### 2.4. Supplement of Short Chain Fatty Acids (SCFAs) Rescued Endothelial Senescence *In Vitro* and *In Vivo*

**Figure 4:**
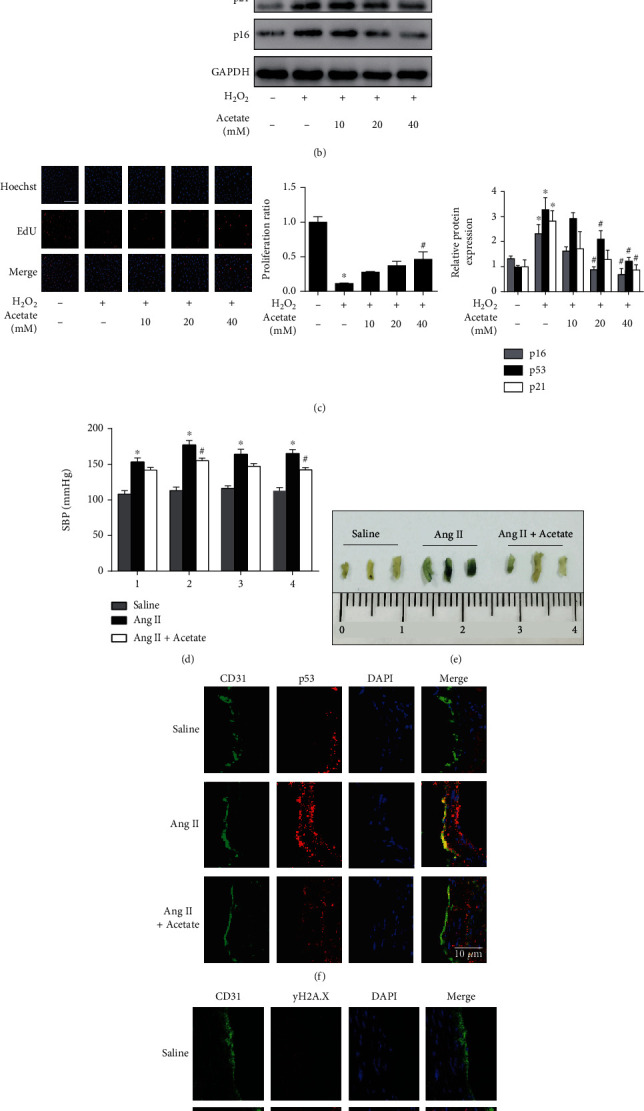
Acetate ameliorated endothelial senescence *in vitro* and *in vivo*. (a–c) Acetate was treated for 24 h in senescent HUVECs induced by H_2_O_2_. (a) SA-*β*-gal staining (scale bar: 50 *μ*m), (b) the protein expression of p53, p21, and p16, and (c) EdU staining (scale bar: 100 *μ*m) were investigated. *n* = 3 ~ 4. Data were presented as means ± SEM. ^∗^*P* < 0.05 vs. control; and ^#^*P* < 0.05 vs. H_2_O_2_. (d–f) Mice were infused with saline or Ang II (1,000 ng/kg/min) using osmotic minipumps for 4 weeks. Acetate (200 mM) was given in drinking water for 4 weeks in the Ang II+acetate group. (d) Mean arterial pressure was measured by tail-cuff plethysmography. *n* = 3. Data were presented as means ± SEM. ^∗^*P* < 0.05 vs. saline; and ^#^*P* < 0.05 vs. Ang II. (e) SA-*β*-gal staining of the inner layer of aortas of mice. *n* = 3. (f) Immunofluorescent staining of p53 was performed in the frozen aortic sections of mice. CD31 represented the endothelial layer. DAPI represented cell nucleus of the vasculature. Merge of CD31 (green) and CPT1A (red) was shown in yellow and indicated the expression of CPT1A in the endothelial layer. *n* = 3. (g) Immunofluorescent staining of *γ*H2A.X was performed in the frozen aortic sections of mice. *n* = 3.

**Figure 5:**
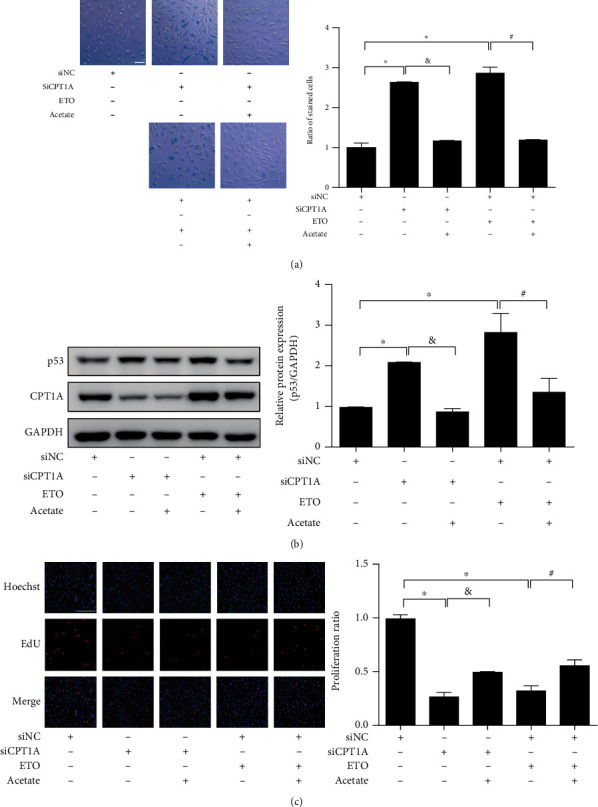
Supplementation of acetate improved endothelial cell senescence induced by si-CPT1A or CPT1A inhibitor ETO. HUVECs were treated with or without siCPT1A (100 nM) or ETO (50 *μ*M) for 24 h, followed by incubation with acetate (40 mM) for 24 h. (a) SA-*β*-gal staining (scale bar: 50 *μ*m) (*n* = 4), (b) Western blot showing the protein expression of p53 (*n* = 6), and (c) EdU staining (scale bar: 100 *μ*m) (*n* = 4) were investigated. Data were presented as means ± SEM. ^∗^*P* < 0.05 vs. control; ^&^*P* < 0.05 vs. siCPT1A; and ^#^*P* < 0.05 vs. ETO.

Different from LCFAs, SCFAs are not dependent on CPT1 for mitochondrial entry, but rather cross the mitochondrial membrane via free diffusion. Thus, SCFAs could bypass the suppression of CPT1A in endothelial senescence and supplement fatty acyl for mitochondrial oxidative pathways [26, 27]. SCFAs include acetic acid, propionic acid, and butyric acid. In this study, acetate or propionate was supplemented to investigate whether or not SCFAs that bypass the reduced CPT1A were able to ameliorate endothelial senescence. In H_2_O_2_-treated senescent endothelial cells, exogenous acetate supplementation prevented the increased expression of SA-*β*-gal, the upregulation of cell cycle repressors, and the proliferation block (Figures 4(a)–4(c)). Consistently, acetate showed antisenescent effect in endothelial cells with CPT1A silencing or inhibition (Figure 5). Similar as acetate, treatment of propionate improved endothelial senescence induced by H_2_O_2_ or CPT1A knockdown/inhibition (Figures S7 and S8).

*In vivo*, acetate was administered to mice continuously infused by a micropump for 28 days. As shown in Figures 4(d)–4(g), treatment of acetate remarkably improved senescence of the endothelial layer of aortas induced by Ang II, as implied by the results of SA-*β*-gal staining and immunofluorescence analysis of p53 and DNA damage foci *γ*-H2AX. Surprisingly, acetate treatment reversed the elevation of blood pressure induced by Ang II infusion (Figure 4(d)).

### 2.5. Fatty Acid Metabolism Regulated Endothelial Senescence via Acetyl-CoA

**Figure 6:**
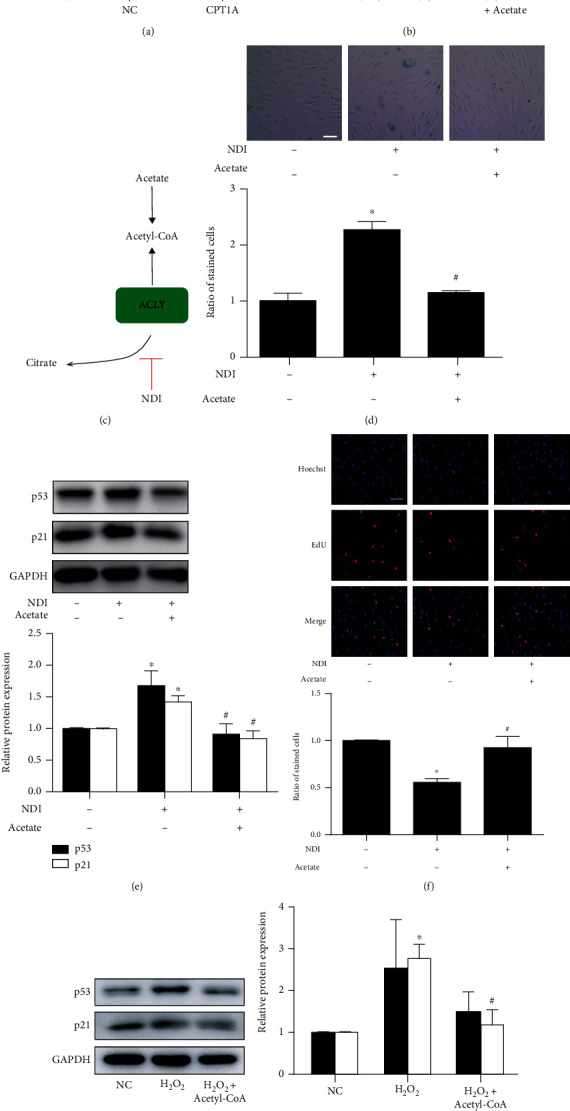
Acetyl-CoA was involved in regulatory effect of fatty acid metabolism in endothelial senescence. (a) The levels of acetyl-CoA in HUVECs transfected with or without CPT1A plasmid. *n* = 3. (b) The levels of acetyl-CoA in HUVECs treated with different concentrations of CPT1A siRNA or nontargeted siRNA control (siNC), with or without acetate (20 mM). *n* = 4. Data were presented as means ± SEM. ^∗^*P* < 0.05 vs. NC or siNC; and ^#^*P* < 0.05 vs. si-CPT1A. (c–e) HUVECs were incubated with ACLY inhibitor NDI (1 *μ*M) for 24 h. Acetate (20 mM) was treated for 24 h. (c) Schematics showing that acetyl-CoA levels can be reduced by the ACLY inhibitor NDI and could be recovered by acetate treatment. (d) SA-*β*-gal staining (scale bar: 50 *μ*m), (e) the protein expression of p53, p21, and p16, and (f) EdU staining (scale bar: 100 *μ*m) were investigated. *n* = 3 ~ 4. Data were presented as means ± SEM. ^∗^*P* < 0.05 vs. control; and ^#^*P* < 0.05 vs. NDI. (g) Acetyl-CoA was treated in HUVECs stimulated with H_2_O_2_, and expression of p53 and p21 was investigated by Western blot. *n* = 3. Data were presented as means ± SEM. ^∗^*P* < 0.05 vs. control; and ^#^*P* < 0.05 vs. H_2_O_2_.

Since acetyl-CoA is the final product of fatty acid metabolism [28], it is possible that the reduction in LCFA oxidation in endothelial senescence leads to changes in acetyl-CoA levels. Indeed, overexpression of CPT1A elevated the intracellular level of acetyl-CoA (Figure 6(a)), whereas deficiency of CPT1A reduced acetyl-CoA production (Figure 6(b)). Replenishment of acetate could rebound the level of acetyl-CoA in endothelial cells with CPT1A silencing (Figure 6(b)).

It is speculated that lack of acetyl-CoA might lead to endothelial senescence and that SCFAs might restrain endothelial senescence through replenishing acetyl-CoA. To verify the role of acetyl-CoA in endothelial senescence, the inhibitor of ATP citrate lyase (ACLY), the enzyme that converts citric acid into acetyl-CoA [29], was used to suppress acetyl-CoA production (Figure 6(c)). ACLY inhibitor NDI-091143 (NDI) augmented senescent marker SA-*β*-gal, accelerated the expressions of p53 and p21, and repressed cell proliferation, suggesting that reduction of acetyl-CoA levels exacerbates senescence of endothelial cells (Figures 6(d)–6(f)). Acetic acid can directly generate acetyl-CoA by acyl-CoA synthetase (ACS) [30]. Therefore, supplementation of acetate could compensate for the depleted acetyl-CoA [7, 31]. Indeed, treatment of acetate reversed NDI-induced senescence in endothelial cells (Figures 6(d)–6(f)).

Moreover, supplementation of acetyl-CoA reversed H_2_O_2_-induced upregulation of senescent markers, further confirming that acetyl-CoA plays a pivotal role in improving endothelial senescence (Figure 6(g)).

### 2.6. Acetylated Protein Profile Was Altered in Senescent Endothelial Cells

**Figure 7:**
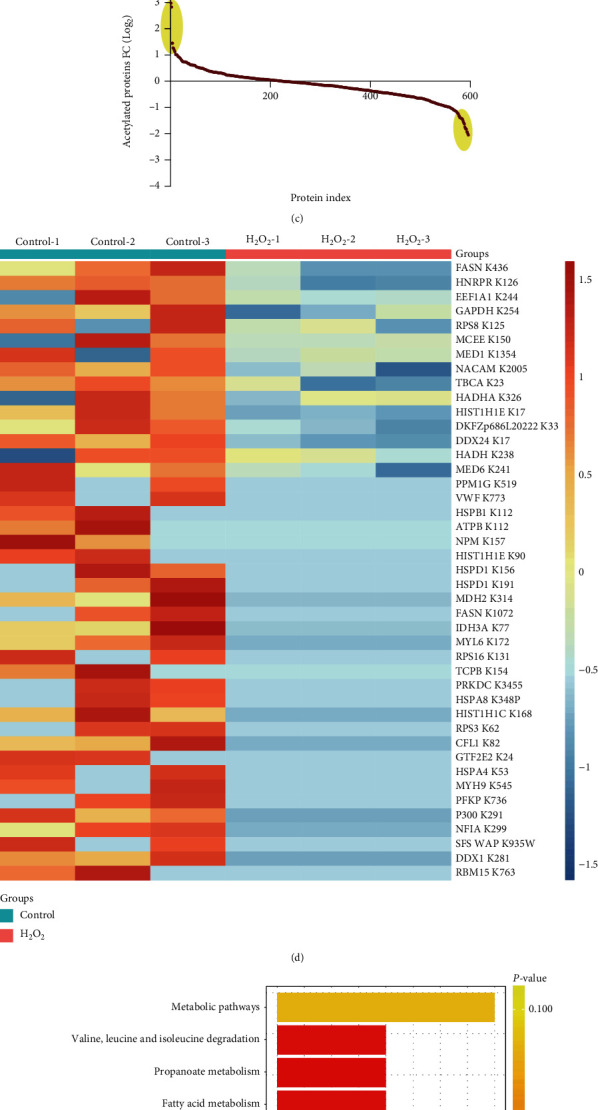
Global proteomic analysis of lysine acetylation in senescent endothelial cells induced by H_2_O_2_. (a) Western blot analysis of total protein acetylation levels. (b) Volcano plot showing the changes of acetylation at intracellular protein lysine sites. (c) Protein index showing the changes of acetylated proteins. (d) Heat map showing the changes of acetylated level at protein lysine sites with statistical significant difference. (e) KEGG pathway analysis of proteins with altered acetylation. *n* = 3.

As the final product of FAO, acetyl-CoA serves as the predominant acetyl donor for lysine acetylation and thereby links metabolism, signaling, and epigenetics [32, 33]. The observations that acetyl-CoA participated in regulation of endothelial senescence thus prompted the hypothesis that acetyl-CoA might affect protein acetylation modification in endothelial senescence. To test this hypothesis, the acetylated proteins were detected through Western blotting using an anti-acetyl lysine antibody. The results demonstrated that most of the detected proteins displayed a declined trend of acetylation level in H_2_O_2_-induced senescent endothelial cells (Figure 7(a)). Moreover, a global lysine acetylome analysis was performed in senescent endothelial cells. Generally, 2706 acetylated proteins were identified, with 1208 validated acetylated-lysine sites. Using ratio > +/−2 and *P* value <0.05 as the screening standard, 40 proteins with 43 acetylated-lysine sites demonstrated a significantly decreased acetylation level in the senescent cells (Figures 7(b)–7(d)). The KEGG pathway analysis revealed that these proteins were closely related to (1) cell energy metabolism, including enzymes involved in fatty acid metabolism such as fatty acid synthetase (FASN), HADHA, and HADH, and that involved in glycolysis such as phosphoglycoside oleate dehydrogenase (GADPH) and platelet-type phosphofructokinase and (2) cell cycle regulation, including DNA-dependent protein kinase catalytic subunit (PRKDC) and 40S ribosomal protein S3/8 (Figure 7(e)). Taken together, these results imply that deficit of acetyl-CoA caused by defect of CPT1A-dependent fatty acid metabolism leads to suppression of protein acetylation which in turn exacerbates cell metabolism and represses cell cycle, finally resulting in endothelial senescence.

## 3. Discussion

The present study provides novel insight into endothelial fatty acid metabolism in the regulation of endothelial senescence. Disturbances in fatty acid metabolism occur during both inducible senescence and replicative senescence in endothelial cells. This conclusion is supported by the following observations: (1) Palm-BSA-stimulated increase of OCR was abrogated in senescent HUVECs; (2) acetyl-CoA, the final product of fatty acid metabolism, was declined; (3) the levels of a series of MCFAs and LCFAs were reduced during endothelial senescence; and (4) proteins associated with fatty acid uptake and mitochondrial entry were downregulated. Usually, the endothelial cells take up MCFAs and LCFAs from the circulation by designated transporters including the FATP and FABP family [34, 35]. The downregulation of FABPpm, FABP4, and FATP6 in either H_2_O_2_-induced senescence model or replicative senescence model indicated a defect of fatty acid uptake, in line with the observations that most of the MCFAs and LCFAs were decreased in senescent endothelial cells. The ACSL family, in concert with FATPs, activates fatty acids destined for beta-oxidation through the process of vectorial acylation [36]. The decrease of ACSL3 mRNA level thus confirms the reduced efficiency in the vectorial acylation of exogenous fatty acids during endothelial senescence. Moreover, the expressions of CPT1 and CPT2 were attenuated, suggesting that the trafficking of fatty acids into mitochondria is repressed. CPT1A, the most abundant isoform of CPT1 in endothelial cells, displayed a dramatic decrease in both replicative and inducible senescent endothelial cells, as well as in the endothelial layer of aortas of SHR and Ang II-infused mice. Considering that CPT1A is the rate-limiting enzyme of FAO [37], the downregulation of CPT1A might act as a crucial determinant facilitating the disturbance of fatty acid metabolism during the development of endothelial senescence. After being transported into mitochondria, acyl-CoA is catalyzed into acetyl-CoA through dehydrogenation, hydration, re-dehydrogenation, and thiolysis. These processes require the dehydrogenases long/medium/short chain 3S-hydroxyacyl-CoA dehydrogenase, the enoyl-CoA hydratase, the dehydrogenase HADH which is responsible for re-dehydrogenation of MCFAs and SCFAs, and mitochondrial trifunctional proteins HADHA and HADHB that exert dehydrogenation, hydration, and thiolysis activities on LCFAs [38]. Intriguingly, the mRNA levels of these enzymes were slightly changed in endothelial senescence models, thus excluding the possibility that abnormality of the oxidation process of fatty acids is involved in endothelial senescence. Since FAO-derived acetyl-CoA helps to sustain the TCA cycle in conjunction with anaplerotic substrates, the repression of fatty acid metabolism might help to explain the decrease of TCA cycle-associated organic acid metabolites and the increase of NAD/NADH ratio. Taken together, these findings support the view that endothelial senescence is accompanied with disruption of fatty acid metabolism.

The present study also attempted to investigate whether or not disorder of fatty acid metabolism facilitates the pathogenesis of endothelial senescence, and improvement of fatty acid metabolism could ameliorate endothelial senescence. Taking into consideration the importance of CPT1A in fatty acid metabolism, CPT1A was knocked down or inhibited to disrupt fatty acid metabolism or was overexpressed to improve FAO. Indeed, CPT1A expression is closely associated with maintaining high FAO level; depletion or inhibition of CPT1A results in FAO deficiency in endothelial cells [7, 11, 39]. Our observations demonstrated that si-CPT1A or CPT1 inhibitor ETO initiated senescence of endothelial cells in a concentration- and time-dependent manner, whereas overexpression of CPT1A could reverse H_2_O_2_-induced senescence. Therefore, these results suggest that maintenance of FAO by CPT1A is important for protecting against endothelial senescence. In addition, exogenous SCFAs were supplemented to bypass CPT1A defect in endothelial senescence. Acetic acid is directly converted into acetyl-CoA by ACS. Propionic acid is catalyzed by ACS to generate propionyl-CoA, followed by conversion into succinyl-CoA, and finally entering TCA cycle to produce acetyl-CoA [40]. Thus, these SCFAs could produce acetyl-CoA, no matter directly or indirectly, to compensate for the deficit in LCFAs metabolism and to rescue fatty acid metabolism [7, 26]. According to our observations, both acetate and propionate exhibited antisenescent effect in endothelial cells stimulated by oxidative stress or with CPT1A knockdown or inhibition. Most importantly, supplement of acetate lowered the blood pressure and alleviated the senescence-related phenotypes in arteries of Ang II-infused mice. These convincing evidences thus prompt the conclusion that rescue of FAO by SCFA supplementation might help to ameliorate endothelial senescence and confer endothelial protection (Figure 7). In fact, our findings about the beneficial effect of SCFAs on endothelial cells are in line with clinical and experimental observations that SCFAs protected from cardiovascular damage and improved cardiovascular health [41–43] and suggest clinical benefit of SCFA treatment in endothelial senescence-related diseases. Overall, these findings hint that improvement of fatty acid metabolism in endothelial cells contributes to maintaining cardiovascular homeostasis and preventing cardiovascular diseases.

The possible mechanisms underlying the regulation of fatty acid metabolism in endothelial senescence are still underexplored. Three possibilities are proposed: (1) fatty acid-derived dNTPs facilitate DNA synthesis and accelerate endothelial cell proliferation, ultimately repressing senescence [7]; (2) NADPH, an intermediate product of FAO, maintains redox homeostasis and prevents endothelial cells from oxidative stress [11]; and (3) FAO-derived acetyl-CoA, which acts as a donor providing acetyl group, might be involved in acetylation modification and epigenetic regulation in endothelial cells to maintain endothelial function [12]. Our study suggests that fatty acid metabolism regulates endothelial senescence via production of acetyl-CoA. The involvement of acetyl-CoA is supported by the following observations: (1) CPT1A overexpression increased acetyl-CoA level, while CPT1A knockdown reduced acetyl-CoA level; (2) suppression of acetyl-CoA production by ACLY inhibitor NDI accelerated senescence of endothelial cells; (3) replenishment of acetyl-CoA by acetate supplementation abolished NDI-induced senescence; and (4) supplementation of acetyl-CoA prevented H_2_O_2_-induced endothelial senescence.

It is still uncertain how acetyl-CoA regulates endothelial senescence. One possibility is that the lack of acetyl-CoA due to FAO metabolic defect might probably alter the acetylated protein profile in senescent endothelial cells. Indeed, acetylation modification may be enzymatically catalyzed by acetyltransferases like p300, but can also be driven nonenzymatically by acetyl-CoA. In most cases, acetyl-CoA is also used by p300 to acetylate histones and nonhistones. Therefore, acetyl-CoA is pivotal for catalyzing acetylation modification. As implied by results of lysine acetylome analysis, those proteins with decreased acetylation level in senescent HUVECs are involved in regulation of cell energy metabolism and cell cycle regulation. The observations of declined acetylation of enzymes related to glycolysis such as GADPH and platelet-type phosphofructokinase might allow speculations that fatty acid metabolism might influence energy homeostasis by coordinating glycolysis, although FAO itself is dispensable for ATP production.

Intriguingly, the relationship between acetyl-CoA, acetylation, and cell senescence seems complicated. In tissues such as skeletal muscle, heart, and adipose tissue, cytoplasmic acetyl-CoA levels are decreased through downregulation of ACLY in response to calorie restriction, to decrease the activity of acetyltransferase p300, finally stimulating prolongevity autophagy [44, 45]. These observations seem to be contradictory to our results that acetyl-CoA prevented endothelial senescence. On the contrary, increasing nuclear acetyl-CoA levels promotes longevity through increased histone acetylation [44, 46] in the hippocampus and other brain regions. According to our results, histone acetylation was not changed according to the whole detected acetyl-lysine profile, thus excluding the possibility that FAO regulates endothelial senescence via epigenetic regulation. It seems that the role of acetyl-CoA in senescence and longevity is diverse in different cellular compartments, different pathological conditions, and even different species [44]. Since our study did not separately investigate the role of FAO-generated acetyl-CoA in the cytoplasm and in the nucleus, the present results do not permit further speculation on the reasons for those apparent discrepancies. Further investigations are needed to elucidate the regulation of acetyl-CoA in the acetylation substrates during endothelial senescence.

To be noted, an increase of intracellular NAD/NADH was observed during H_2_O_2_-induced endothelial senescence. Since NAD^+^ is required for the activation of sirtuins, the class III histone deacetylase family, it might lead to the speculation that sirtuins are activated to coordinate global protein acetylation together with acetyl-CoA. However, a large amount of evidence indicates that the expressions of several sirtuin family members, including SIRT1 [47], SIRT2 [48], SIRT3 [49], and SIRT6 [50], are significantly decreased during endothelial senescence. Thus, it challenged the possibility that the increased NAD^+^ is able to compensate the downregulation of sirtuins. Unlike in heart and skeletal muscle, where global protein acetylation is increased with aging mainly due to decreased NAD^+^ levels with aging decreasing sirtuin activity [51], the change of global protein acetylation in endothelial cells might be affected by a complex regulatory network containing acetyl-CoA, NAD^+^, sirtuins, and acetylases like p300.

As a limitation of the present study, the involvement of fatty acid-derived dNTPs and NADPH in the regulation of endothelial senescence cannot be excluded, since endothelial senescence is closely associated with cell cycle regulation and oxidative stress. Additionally, CPT1A endothelial cell-specific knockout/transgenic mice were not used to prove the in vivo effect.

**Figure 8:**
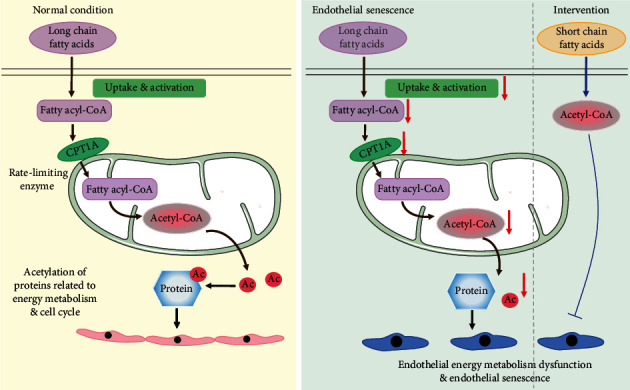
Schematics showing the conclusion of the present study. In normal condition, vascular endothelial cells rely on fatty acid oxidation to produce acetyl-CoA, which facilitates protein acetylation modification. However, a disturbed fatty acid profile and suppressed FAO occur in senescent endothelial cells, which is characterized by CPT1A deficiency. FAO impairment by knockdown or inhibition of CPT1A facilitates the development of endothelial senescence via repression of acetyl-CoA-induced acetylation modification, whereas improvement of fatty acid metabolism by CPT1A overexpression ameliorates endothelial senescence. Moreover, supplementation of short chain fatty acids ameliorates endothelial senescence.

In conclusion, the present study identifies a disturbed fatty acid profile and suppressed FAO in senescent endothelial cells (Figure 8). FAO impairment by knockdown or inhibition of CPT1A facilitates the development of endothelial senescence, whereas improvement of fatty acid metabolism by CPT1A overexpression or SCFA supplementation ameliorates endothelial senescence. Mechanistically, fatty acid metabolism regulates endothelial senescence via acetyl-CoA-induced acetylation modification. Therefore, therapeutic strategies targeting endothelial fatty acid metabolism might shed new lights on the treatment of cardiovascular diseases associated with endothelial senescence and vascular aging.

## 4. Materials and Methods

### 4.1. Cell Culture

Human umbilical cords were collected from the First Affiliated Hospital of Sun Yat-sen University in Guangzhou, China. HUVECs were isolated and cultured as previously described [52]. Briefly, endothelial cells from the vein of human umbilical cord were digested with trypsin and cultured in endothelial cells medium (ECM, ScienCell, San Diego, CA, USA) in a humidified atmosphere of 5% CO_2_ at 37°C.

### 4.2. Endothelial Cell Senescent Models and Treatments

Replicative endothelial senescence model was induced by population doublings in cell culture. Cells at 13~16 passages were considered as “senescent,” while cells at passage 3~6 were regarded as the “young” control. For H_2_O_2_-induced endothelial senescence model, HUVECs were treated with 100 *μ*M H_2_O_2_ for 1 h following culture with 20% ECM in Medium 199 for 48 h. In some cases, HUVECs were incubated with the CPT1 inhibitor etomoxir (Macklin, Shanghai, China) at the concentration of 1 *μ*M, 10 *μ*M, and 50 *μ*M for 24 h or 48 h. The ACLY inhibitor NDI-091143 (Macklin, Shanghai, China) was treated at a final concentration of 1 *μ*M for 48 h. Acetate (40 mM), propionate (4 mM), and acetyl-CoA sodium salt (1 mM) were obtained from Macklin (Shanghai, China) and were incubated with cells for 24 h after stimulating by H_2_O_2_ or etomoxir for 24 h.

### 4.3. Animal Studies

Animal procedures used in this study were in accord with institutional guidelines and were approved by Laboratory Animal Center of the Sun Yat-sen University. Male C57BL/6 mice (purchased from Guangdong GemPharmatech Company) were randomly divided into 3 groups as follows: (i) control group, (ii) Ang II group, and (iii) Ang II+acetate (Aladdin) group. At the age of 8 weeks, the mice were infused with saline or Ang II (1,000 ng·kg^−1^·min^−1^) using osmotic minipumps (Model 2004; Alzet) for 4 weeks. At the same time, mice of the Ang II+acetate group were supplied with 200 mM acetate in drinking water for 4 weeks, refreshed three times per week. Systolic blood pressure (BP) was measured by tail-cuff plethysmography (BP-2010A; Softron Biotechnology) every week. The body weights of the mice were weighed on a scale every three days. All the mice were caged in a temperature- and humidity-controlled room with a 12 h light/dark cycle and fed a standard chow diet and clean water. Twelve-week-old male Wistar Kyoto (WKY) rats and Spontaneous Hypertension Rat (SHR) were purchased from the Charles River Laboratories (Beijing, China). Blood pressure was measured at room temperature via carotid artery cannulation after anaesthetization. After being sacrificed, aortas were dissected in oxygenated ice-cold Kreb's solution and then quickly frozen in liquid nitrogen. The frozen aortas were cut into 5 *μ*M frozen sections by Servicebio Company.

### 4.4. Transfection of CPT1A siRNA or Plasmid

The siRNAs used to knock down CPT1A in HUVECs were synthesized by GenePharma (Suzhou, China). CPT1A plasmid was extracted with Tiangen kit (Beijing, China) and transfected with jetOPTIMUS (Polyplus-transfection SA, NY, USA). The nontargeted siRNA was served as negative control (siNC).

### 4.5. Western Blot Analysis

For Western blot analysis, HUVEC lysates were collected with RIPA Buffer (Beyotime, Shanghai, China) and protein concentration was determined using a BCA protein assay kit (Thermo Fisher, Rockford, IL, USA). Proteins were separated by electrophoresis with 10%-12% SDS polyacrylamide gel and then were transferred to polyvinylidene difluoride (PVDF) membranes (Millipore, Billerica, MA, USA). Following blocking at room temperature with 5% skimmed milk for 1 h, the membranes were incubated with the indicated primary antibodies at 4°C overnight. After that, membranes were washed in Tris-buffered saline tween (TBST) and latter incubated with secondary anti-rabbit or anti-mouse antibody. The bound secondary antibody was visualized by chemiluminescence using ECL™ Western Blotting Detection Reagent (GE Healthcare). The rabbit polyclonal anti-CPT1A, rabbit polyclonal anti-p53, and rabbit polyclonal anti-p21 were obtained from Proteintech Group (IL, USA), and the rabbit polyclonal anti-p16 was purchased from Abcam (Cambridge, UK).

### 4.6. Quantitative Real-Time Polymerase Chain Reaction

Total RNA from cultured HUVECs were extracted using TRIzol reagent (Takara Biotechnology, Dalian, China) according to the RNA extraction protocol. Complementary DNA was prepared using Revert Aid First Strand cDNA Synthesis Kit (Thermo, MA, USA). The real-time RT-PCR analysis was performed using 2× SYBR-Green qPCR Mix (Dongsheng Biotech, Guangzhou, China) with LightCycler 480II (Roche, Basel, Switzerland). Data were analyzed using the comparative cycling threshold (*^ΔΔ^*Ct) method. The GAPDH was used as an internal control, and human-specific primers were synthesized and purified by Sangon (Shanghai, China). The primer sequences are shown in Supplementary Table 1.

### 4.7. Senescence-Associated-*β*-Galactosidase (SA-*β*-Gal) Staining

HUVECs were seeded on a fibronectin-coated culture 48-well plate. The freshly dissected aortas were mounted on clean plates. SA-*β*-gal was stained by using a SA-*β*-gal detection kit (Beyotime Biotechnology, Shanghai, China). The cells or aortas were immersed in the staining solution and incubated at 37°C for 12 h. Cells with blue staining were suggested to be SA-*β*-gal-positive cells.

### 4.8. Immunofluorescence Staining

Frozen sections were fixed with 5% paraformaldehyde for 15 minutes at room temperature. After rinsing, the sections were treated with 0.5% Triton X-100 to permeate into the cell membrane. Following washing with PBS 3 times, the sections were blocked with 5% goat serum in PBS for 1 h at room temperature. Anti-CPT1A, anti-p53, and anti-CD36 primary antibodies were diluted in 5% goat serum and incubated overnight at 4°C. After rinsing, sections were revealed by a combination of anti-mouse and anti-rabbit antibody conjugated to Alexa Fluor 488 and 594 at 1 : 200. Fluorescent images were captured by a laser scanning ultrahigh-resolution microscope (FV 3000, Olympus), and relative fluorescence intensity was calculated using ImageJ software.

### 4.9. EdU Staining

Since senescent cells undergo cell cycle arrest, the defect of proliferation ability suggests cellular senescence. Thus, the proliferation ability of HUVECs was determined using the EdU (5-ethynyl-2′-deoxyuridine) staining kit (RiboBio Co., Ltd., Guangzhou, China) according to the protocol. Images were acquired by cell auto imaging system (EVOS FL Auto, Life Technologies, New York, USA). The EdU-positive cells were shown red. Ratio of proliferating cells was normalized to the total cell numbers stained with Hoechst (blue).

### 4.10. Measurements of Fatty Acid Metabolism in HUVECs by Seahorse Assay

The cell's ability to metabolize fatty acid was assessed by Seahorse XF96 extracellular flux analyzer (Agilent, CA, USA). HUVECs were seeded 15,000 per well on XF96 cell culture microplate. After attaching to the plate, the cells were incubated with substrate-limited medium (according to Manufacturer's protocol) for 4 h. For FAO assessment, the cells were incubated with FAO assay buffer in non-CO_2_ incubator for 45 minutes, followed by addition of palmitate-conjugated bovine serum albumin (Palm-BSA) or BSA. Oxygen-consumption rate (OCR) was to assess the cell's FAO ability.

### 4.11. Metabolomics Detecting Fatty Acid Profile and TCA Metabolites in HUVECs

The treated cells were washed with PBS and 0.9% NaCl solution, digested with trypsin, centrifuged at 800*g*, 4°C, and then frozen with liquid nitrogen and stored in -80°C. Cells retrieved from −80°C storage were ground into fine powder in liquid nitrogen. Powder (60 mg) was vortexed in a 1 mL solution of methanol/acetonitrile/H_2_O. After sonication for 30 min on ice, the mix was stored at -20°C for 1 h to precipitate proteins. The mix was centrifuged for 15 min (13,000 rpm, 4°C) and dried by a vacuum drying system. A targeted metabolic analysis was performed using an LC-MS/MS system. The dried metabolites were dissolved in 100 *μ*L of acetonitrile/H_2_O (1 : 1, *v*/*v*) and centrifuged (13,000 rpm) for 15 min. Electrospray ionization was conducted with an Agilent 1290 Infinity chromatography system and AB Sciex QTRAP 5500 mass spectrometer. NH4COOH (15 mM) and acetonitrile were used as mobile phases A and B, respectively. A binary solvent gradient was used as follows: A, NH_4_COOH; B, 0-18 min at 90% to 40% acetonitrile; 18-18.1 min at 40% to 90% acetonitrile; and 18.1-23 min at 90% acetonitrile. The LCMS/MS was operated in the negative mode under the following conditions: source temperature, 450°C; ion source gas 1, 45; ion source gas 2, 45; curtain gas, 30; and ion spray voltage floating (ISVF), -4500 V. Metabolites from six individual samples from one group were detected. Metabolomics was performed by Shanghai Applied Protein Technology Co. Ltd. (Shanghai, China).

### 4.12. Proteomic Profiling of Lysine Acetylation

HUVECs were seeded in 100 mm culture dishes for treatment. After that, the cells were washed with precooled PBS, lysed, and collected by urea lysate. The cell lysate was cleaved into proteins by trypsin. The acetylated peptides were enriched using the PTMScan® Pilot Acetyl-Lysine Motif Kit (Cell Signaling Technology, Massachusetts, USA). The acetylated lysine motifs were detected by LC/MS/MS (Nanoflow HPLC: EASY-nLC1000; Q-Exactive Mass: Thermo Finnigan). The mass-spectrometric data was analyzed by a quantitative proteomics software MaxQuant. The proteomics was performed by Shanghai Applied Protein Technology Co. Ltd. (Shanghai, China).

### 4.13. Acetyl-CoA Measurement

The cells were collected into a centrifuge tube. 5∗10^5^ cells were dissolved in 100 *μ*L extraction solution. After sonication for 30 min on ice, the extraction solution was centrifuged at 10,000*g* for 10 min to collect supernatant. The supernatant was placed on ice to be tested. Acetyl-CoA levels were measured using an Acetyl Coenzyme A Content Assay Kit (BOXBIO, AKFA019U-2, China) following the manufacturer's protocol.

### 4.14. Statistical Analysis

Statistical analysis was performed by unpaired Student's *t*-test for control and treatment comparisons or by one-way ANOVA analysis with *Bonferroni post hoc* test for multiple comparisons. Statistical analyses were performed using GraphPad Prism 8.0 (GraphPad Software, La Jolla, CA, USA). Results were presented as Mean ± SEM. *P* value <0.05 was considered to indicate statistically significant differences.

## Data Availability

The datasets used and/or analyzed during the current study are available from the corresponding author on reasonable request.

## Abbreviations

HUVECs: Human umbilical vein endothelial cells
FAO: Fatty acid oxidation
CPT1A: Carnitine palmitoyl transferase 1A
SCFAs: Short chain fatty acids
Acetyl-CoA: Acetyl-coenzyme A
EndoMT: Endothelial-to-mesenchymal transition
NADPH: Nicotinamide adenine dinucleotide phosphate
TCA: Tricarboxylic acid
dNTP: Deoxynucleotide triphosphate
OCR: Oxygen-consumption rate
Palm-BSA: Palmitate-conjugated bovine serum albumin
MCFAs: Medium chain fatty acids
LCFAs: Long chain fatty acids
DHA: Docosahexaenoic acid
FATP: Fatty acid transport protein
FABP: Fatty acid-binding protein
ACSL: Acyl-CoA synthetase of LCFAs
ETO: Etomoxir
SA-*β*-gal: Senescence-associated-*β*-galactosidase
ACLY: ATP citrate lyase
ACS: Acyl-CoA synthetase
GADPH: Phosphoglycoside oleate dehydrogenase.

## Supplementary Materials

Supplementary Materials Supplementary Materials Figure S1: the mRNA level of fatty acid metabolism enzymes was altered in senescent endothelial cells. Figure S2: CPT1A was the principle CPT1 subtype expressed in the endothelial cells. Figure S3: endothelial senescence was obvious in the aortas of SHRs, indicating that SHR can be used as an in vivo model of endothelial senescence. Figure S4: screening of CPT1A siRNA sequences by Western blot. Figure S5: CPT1 inhibitor ETO facilitated endothelial senescence in a dose- and time-dependent manner. Figure S6: CPT1A expression was remarkably upregulated after transfection with the Flag-labeled CPT1A plasmid. Figure S7: propionate ameliorated H_2_O_2_-induced endothelial cell senescence. Figure S8: exogenous supplementation of propionate improved endothelial cell senescence induced by CPT1A depletion or inhibition. Table S1: specific primer sequences for RT-PCR.
