# Supplementary material for: Disturbance of Fatty Acid Metabolism Promoted Vascular Endothelial Cell Senescence via Acetyl-CoA-Induced Protein Acetylation Modification
Source: Oxid Med Cell Longev. 2022 Aug 10;2022:1198607. doi: 10.1155/2022/1198607 (PMC9385365; doi:10.1155/2022/1198607)
Supplement: Supplementary Materials — Figure S1: the mRNA level of fatty acid metabolism enzymes was altered in senescent endothelial cells. Figure S2: CPT1A was the principle CPT1 subtype expressed in the endothelial cells. Figure S3: endothelial senescence was obvious in the aortas of SHRs, indicating that SHR can be used as an in vivo model of endothelial senescence. Figure S4: screening of CPT1A siRNA sequences by Western blot. Figure S5: CPT1 inhibitor ETO facilitated endothelial senescence in a dose- and time-dependent manner. Figure S6: CPT1A expression was remarkably upregulated after transfection with the Flag-labeled CPT1A plasmid. Figure S7: propionate ameliorated H2O2-induced endothelial cell senescence. Figure S8: exogenous supplementation of propionate improved endothelial cell senescence induced by CPT1A depletion or inhibition. Table S1: specific primer sequences for RT-PCR. [file 1198607.f1.docx]

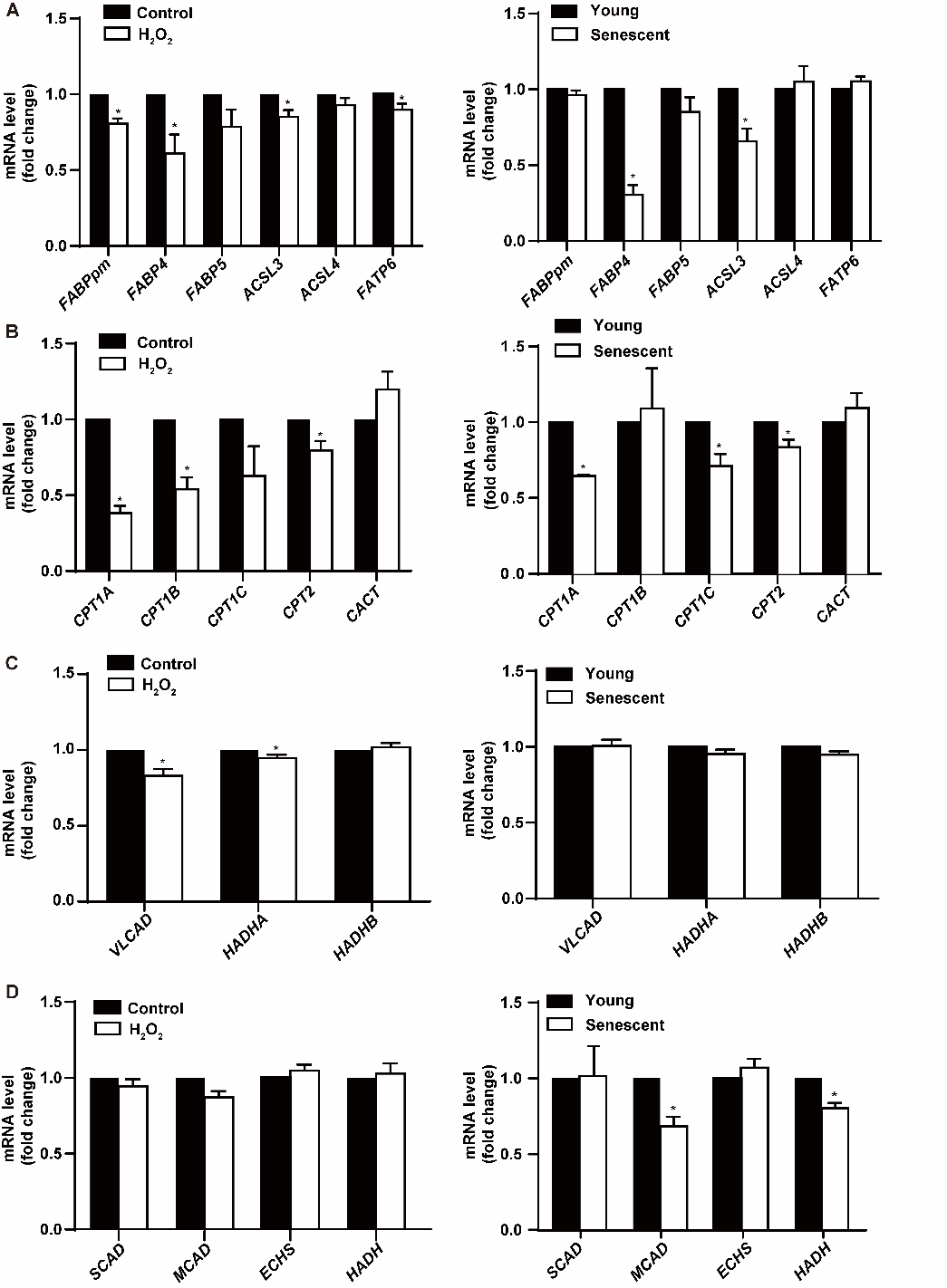


**Supplementary Fig. 1 The mRNA level of fatty acid metabolism enzymes was altered in senescent endothelial cells.** The mRNA expressions of **(A)** enzymes related to fatty acids transport, **(B)** proteins that contribute to fatty acid import into mitochondria, **(C)** enzymes related to oxidation of long chain fatty acyl-CoA, and **(D)** enzymes related to oxidation of short chain fatty acyl-CoA, were measured by quantitative RT-PCR in H_2_O_2_-induced HUVECs senescence model and replicative senescence model. n=4~7. Data were presented as means ± SEM. **P* < 0.05 vs. Control/Young.

**
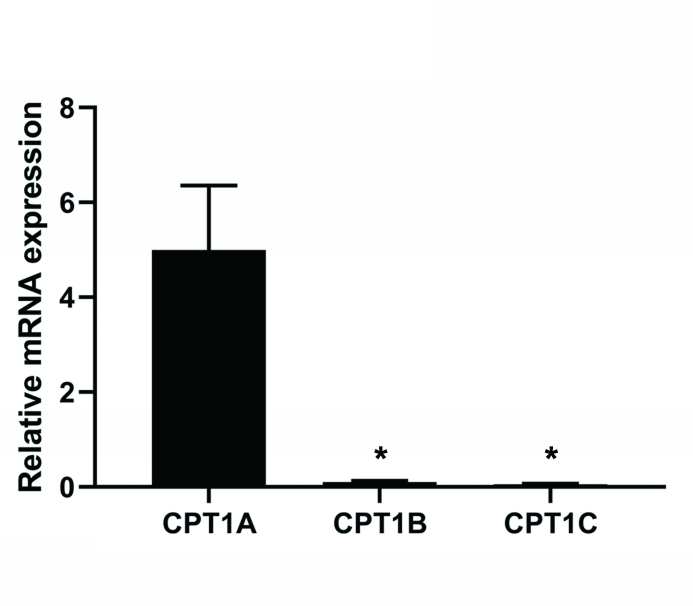
**

**Supplementary Fig. 2 CPT1A was the principle CPT1 subtype expressed in the endothelial cells.** RT-PCR was used to detect the abundance of CPT1 subtypes (CPT1A, CPT1B, CPT1C) in HUVECs. n = 4. **P* < 0.05 as compared to the CPT1A.


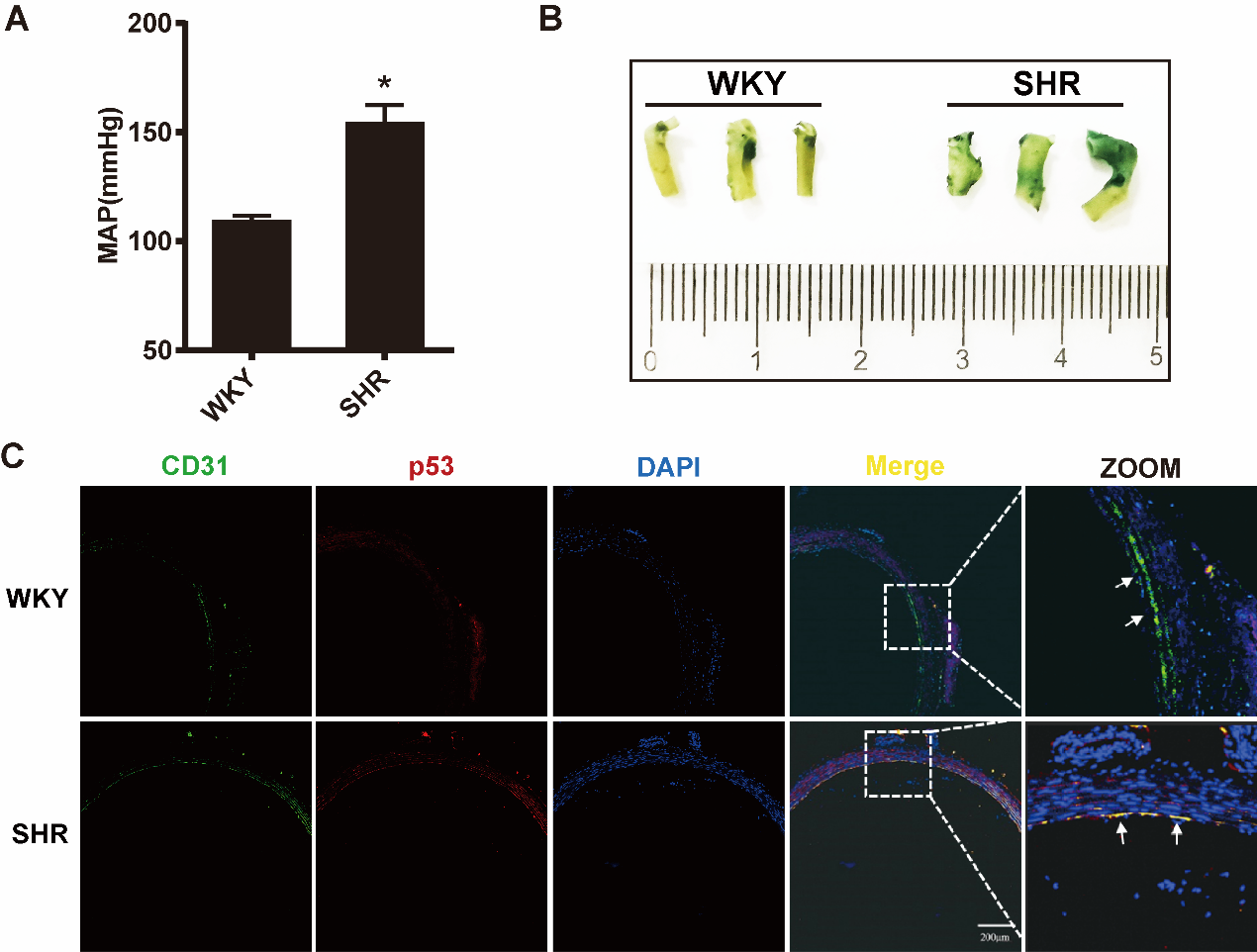


**Supplementary Fig. 3 Endothelial senescence was obvious in the aortas of SHRs, indicating that SHR can be used as an *in vivo* model of endothelial senescence. (A)** Mean arterial pressure was measured via carotid artery cannulation. n=4. Data were presented as means ± SEM. **P* < 0.05 vs. WKY. **(B)** SA-β-gal staining of the inner layer of aortas of SHR and WKY. n=3. **(C)** Immunofluorescent staining of p53 was performed in the frozen aortic sections of SHRs and WKYs. CD31 represented the endothelial layer. DAPI represents cell nucleus of the vasculature. n=4.


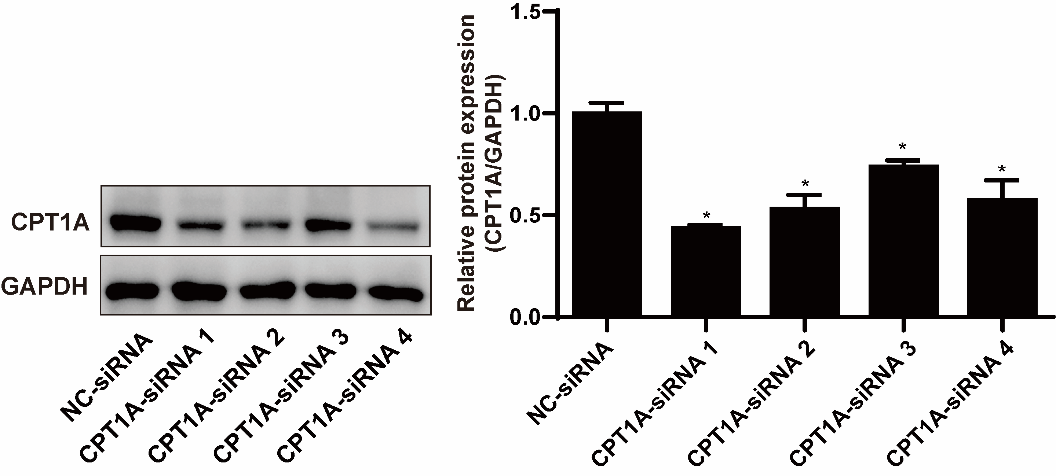


**Supplementary Fig. 4 Screening of CPT1A siRNA sequences by Western blot.** CPT1A-siRNA 1 was detected with the highest silencing efficacy and used for the following experiments. The non-targeted siRNA was served as negative control (NC-siRNA). **P* < 0.05 as compared to the NC-siRNA group. n = 4.


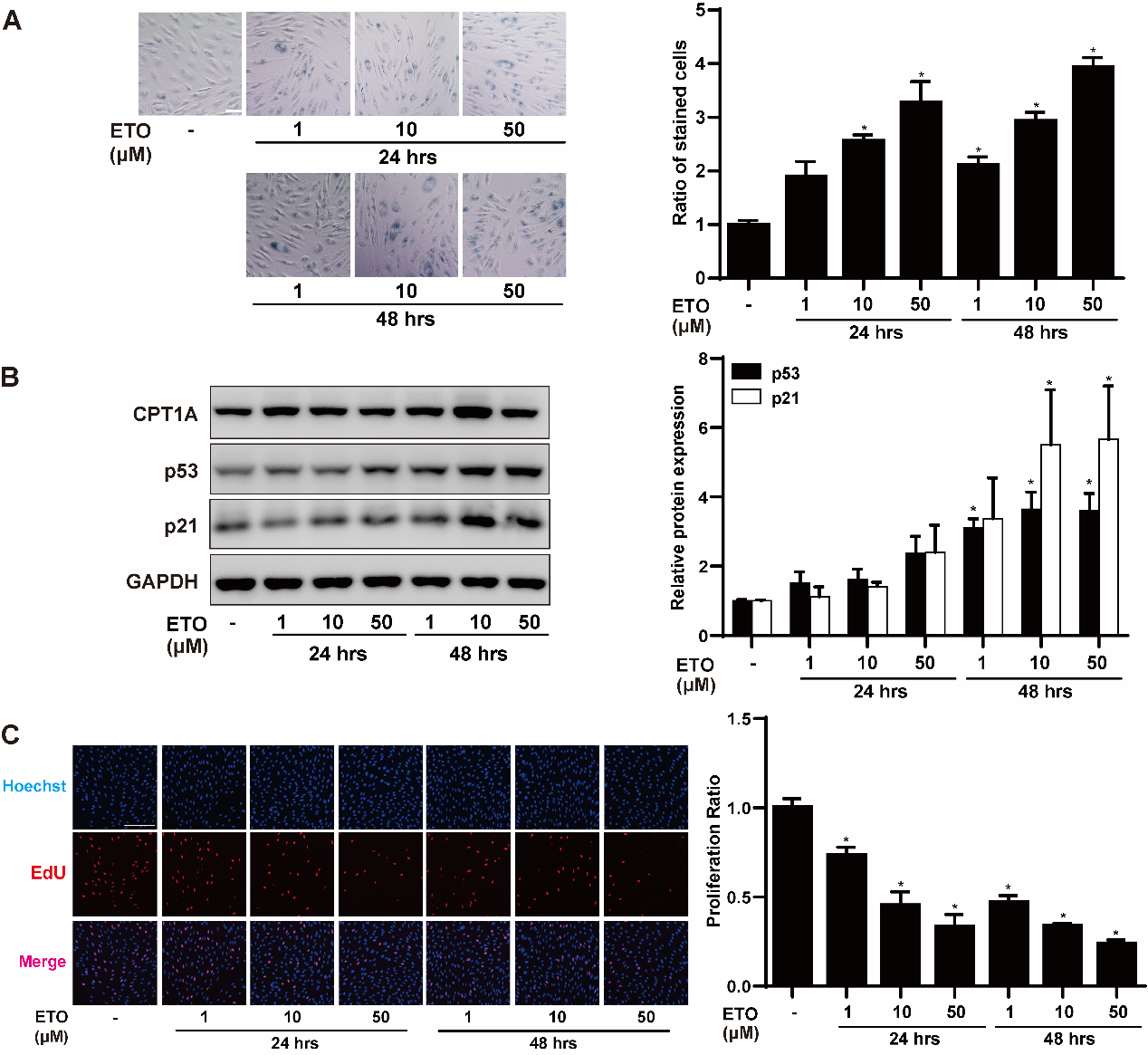


**Supplementary Fig. 5 CPT1 inhibition facilitated endothelial senescence.** HUVECs were incubated with or without different concentrations of CPT1 inhibitor ETO for 24 h or 48 h. **(A)** SA-β-gal staining, scale bar: 50 μm, n=4. **(B)** Western blot showing the protein expression of p53 and p21, n=3. **(C)** EdU staining showing the ratio of proliferating endothelial cells, scale bar: 100 μm, n=4. Data were presented as means ± SEM. **P* < 0.05 vs. Control.


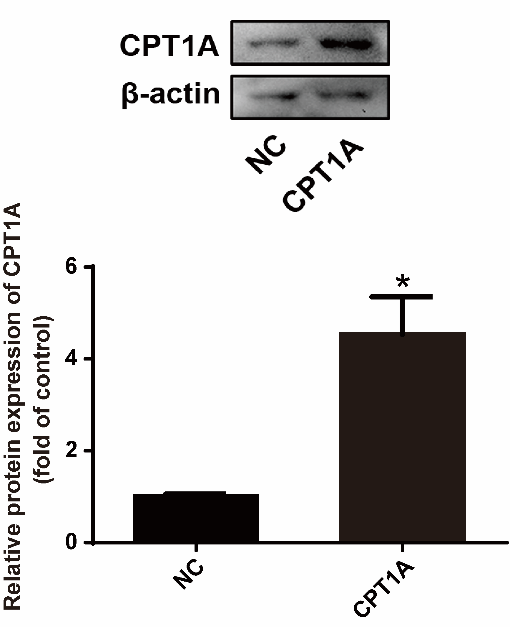


**Supplementary Fig. 6 CPT1A expression was remarkably upregulated after transfection with the Flag-labeled CPT1A plasmid**

HUVECs were transfected with or without Flag-labeled CPT1A plasmid. Western blot showing the protein expression of CPT1A. n=3. Data were presented as means ± SEM. **P* < 0.05 vs. NC.


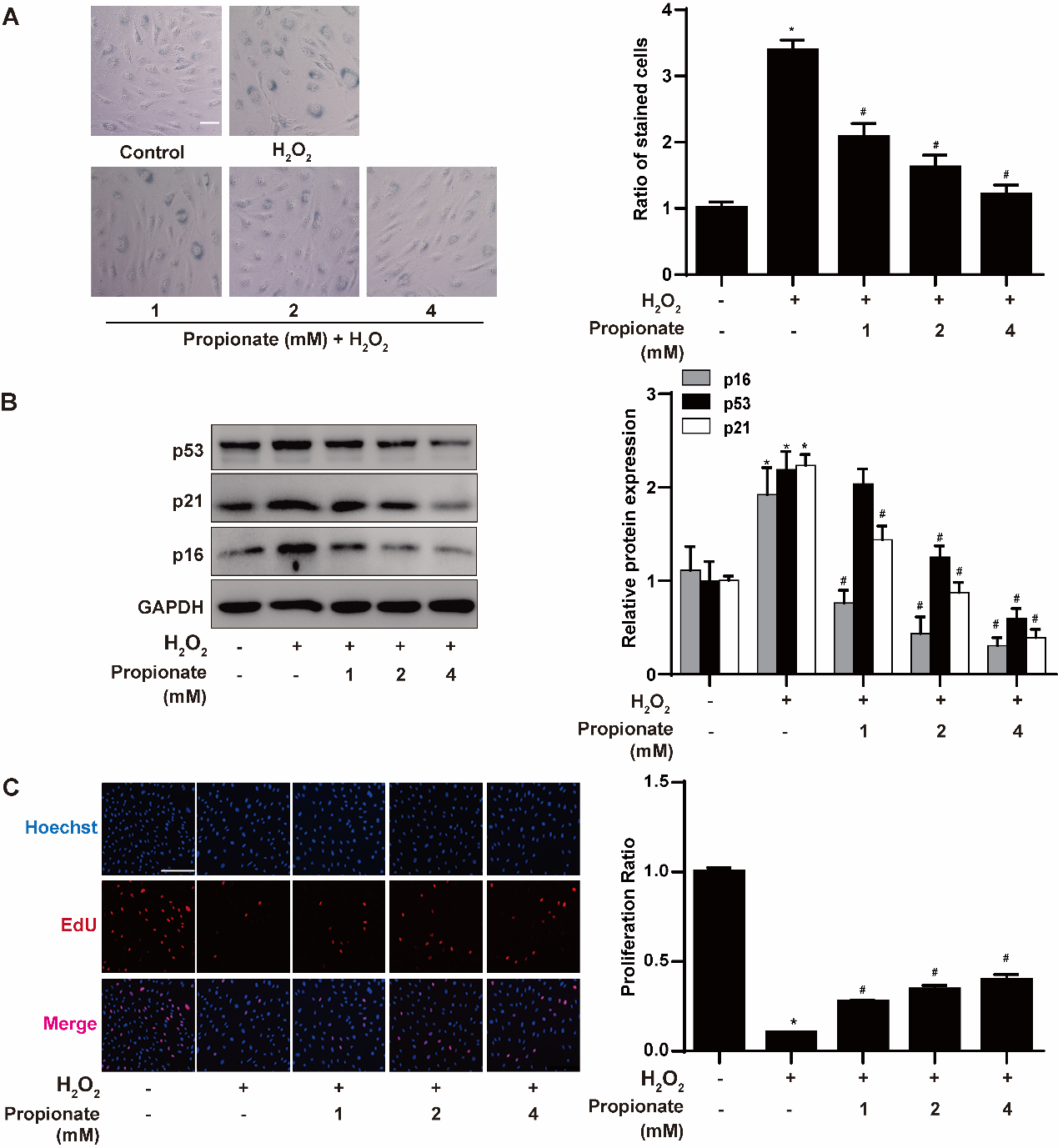


**Supplementary Fig. 7 Propionate ameliorated H_2_O_2_**-**induced endothelial cell senescence.** HUVECs were treated with H_2_O_2_, followed by incubation with different concentrations of propionate for 24 h. **(A)** SA-β-gal staining (scale bar: 50 μm), **(B)** Western blot showing the protein expression of p53, and **(C)** EdU staining showing the ratio of proliferating endothelial cells (scale bar: 100 μm), were investigated. n=3~4. Data were presented as means ± SEM. **P* < 0.05 vs. Control; and ^#^*P* < 0.05 vs. H_2_O_2_.


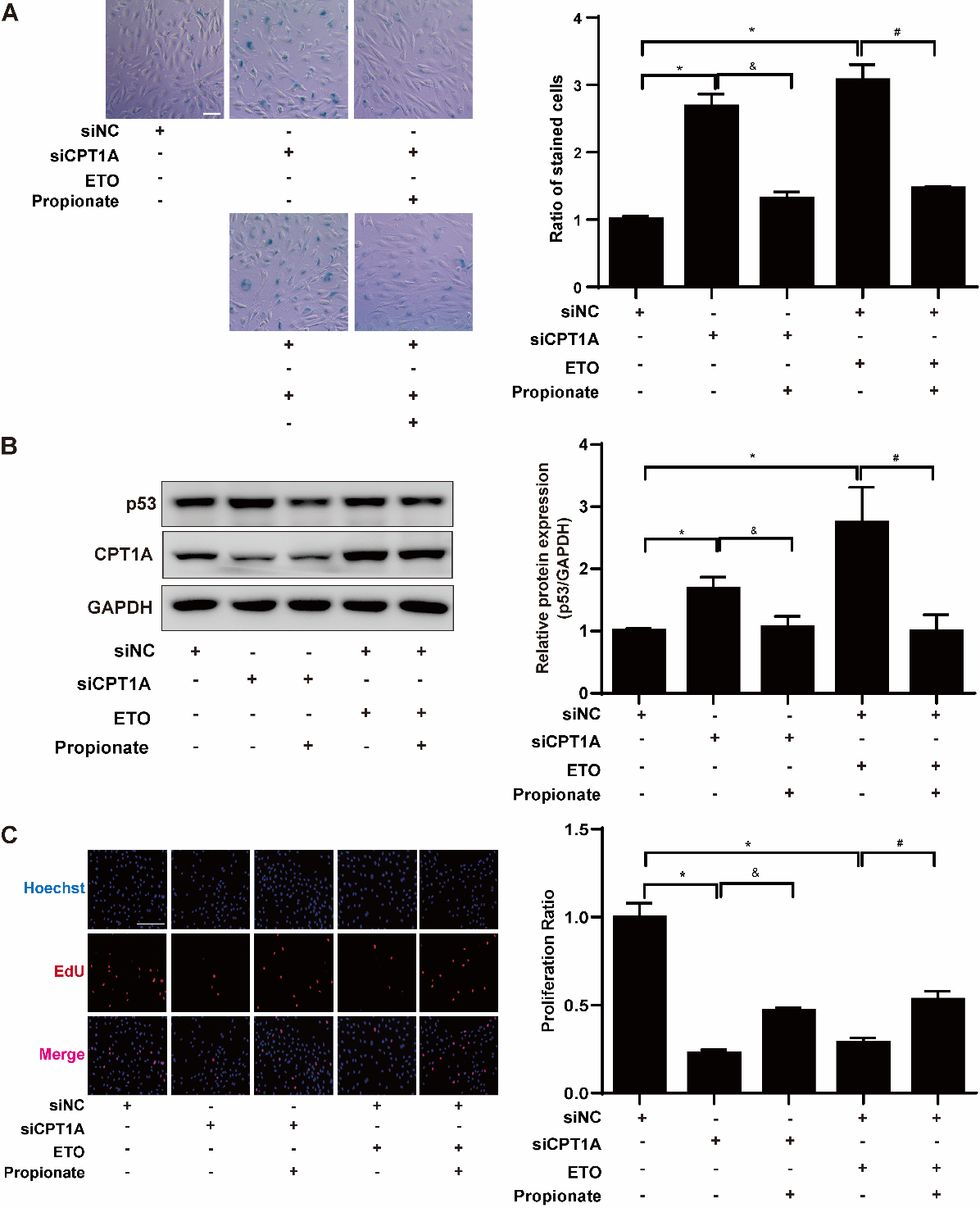


**Supplementary Fig. 8 Exogenous supplementation of propionate improved endothelial cell senescence induced by CPT1A depletion or inhibition.** HUVECs were treated with or without siCPT1A (100 nM) or CPT1 inhibitor ETO (50 μM) for 24 h, followed by incubation with propionate (4 mM) for 24 h. **(A)** SA-β-gal staining (scale bar: 50 μm), **(B)** Western blot showing the protein expression of p53, and **(C)** EdU staining showing the ratio of proliferating endothelial cells (scale bar: 100 μm), were investigated. n=4~5. Data were presented as means ± SEM. **P* < 0.05 vs. Control; ^&^*P* < 0.05 vs. siCPT1A; and ^#^*P* < 0.05 vs. ETO.

**Supplementary Table 1. Specific primer sequences for RT-PCR**

| Target Genes | Sequence5’-3’ | |
| --- | --- | --- |
| GAPDH | Forward | GGTGGTCTCCTCTGACTTCAACA |
|  | Reverse | GTTGCTGTAGCCAAATTCGTTGT |
| FABP4 | Forward | AAACTGGTGGTGGAATGCGT |
|  | Reverse | GCGAACTTCAGTCCAGGTCA |
| FABP5 | Forward | TGAAGGAGCTAGGAGTGGGAA |
|  | Reverse | TCTGCCATCAGCTGTGGTTT |
| FABPpm | Forward | CTGGTGGACCCATGTGGAAA |
|  | Reverse | TGCACAGTGACAAACTTGCG |
| FATP6 | Forward | TGTTGAGTTGGGTGCCACTT |
|  | Reverse | CCAATTGCCAAACGCACCTT |
| ACSL3 | Forward | CCGGCGTAGCGATACAGAAT |
|  | Reverse | AGCTTCTGAGGGTGGCAAAT |
| ACSL4 | Forward | GCGGCTTTTTCTCTGGCCTC |
|  | Reverse | GTTGCTGTAGCCAAATTCGTTGT |
| CPT1A | Forward | CAAACTGGACCGGGAGGAAA |
|  | Reverse | TGTGCTGGATGGTGTCTGTC |
| CPT1B | Forward | GACAGGAGTGAACCCGAGC |
|  | Reverse | GAGGATGCCATTCTTGATGCG |
| CPT2 | Forward | CAGCAGATGATGGTTGAGTGC |
|  | Reverse | GTCAAAGCCCTGGCCCATT |
| CACT | Forward | TCTCGATTCCAGACTGCACC |
|  | Reverse | GGGGTGGCCCAATTAAGGAA |
| VLCAD | Forward | GGAAGCTCGCGGCTCA |
|  | Reverse | AAGGACTTAGATTCCGCCTTGG |
| HADHA | Forward | TCAACATGTTAGCCGCTTGC |
|  | Reverse | ATGGCAACCTCAAGTCCTCC |
| HADHB | Forward | CGGACGTCAGCCAAGATTCC |
|  | Reverse | GCCCATTTTGATGCAGTGGG |
| SCAD | Forward | TACCTGGGGCCCATCTTGAA |
|  | Reverse | ATCACTGCCGTTCCCTGGTT |
| MCAD | Forward | CGGGGTTCGGGCGATG |
|  | Reverse | CTGCTGTTCGGTGAACTCAAA |
| ECHS | Forward | ATCTATGCCGGTGAGAAGGC |
|  | Reverse | GACAAGACCTGCTTGCTTGG |
| HADH | Forward | ACGTGACGGTCATCGGC |
|  | Reverse | TCGCCGGCCTTAAGGTTTTC |

RT-PCR reverse transcription polymerase chain reaction, *GAPDH* glyceraldehyde 3-phosphate dehydrogenase, *FABP* fatty acid binding protein, *FABPpm* PM-associated, *FATP* fatty acid transport protein, *ACSL* long chain acyl-CoA synthetase, *CPT* carnitine palmitoyl transferase, *CACT* carnitine acylcarnitine translocase, *HADH-A/B* mitochondrial trifunctional protein α/β subunit, VL-/M-/S-CAD very long-/medium-/short-chain acyl-CoA dehydrogenase, *ECHS* enoyl-CoA hydratase, *HADH* 3S-hydroxyacyl-CoA dehydrogenase.
